# Combined obesity- and lipid-related indices are associated with hypogonadism in Chinese male patients with type 2 diabetes: a cross-sectional study

**DOI:** 10.3389/fendo.2023.1319582

**Published:** 2024-01-08

**Authors:** Yongzhuo Yu, Yunyang Wang, Lili Xu, Wenxuan Li, Yangang Wang

**Affiliations:** Department of Endocrinology, the Affiliated Hospital of Qingdao University, Qingdao, China

**Keywords:** hypogonadism, type 2 diabetes mellitus, visceral adiposity index, Chinese visceral adiposity index, triglyceride glucose index, lipid accumulation product

## Abstract

**Background:**

There is insufficient attention to hypogonadism in Chinese males with type 2 diabetes mellitus (T2DM). We evaluated the relationship between Combined obesity- and lipid-related indices [Visceral Adiposity Index (VAI), Chinese Visceral Adiposity Index (CVAI), Triglyceride Glucose Index (TyG) and Lipid Accumulation Product (LAP)] with total testosterone (TT) and analyzed the predictive capability of the respective cut-off values.

**Methods:**

We recruited 958 hospitalized male patients with T2DM at the Affiliated Hospital of Qingdao University, collected baseline data and four calculated indices, and obtained their dominance ratio (OR) and corresponding 95% confidence intervals (CI) with TT by multivariate logistic regression. Receiver operating characteristic (ROC) curves were then used to determine cutoff values in predicting hypogonadism (TT< 12 nmol/L), and we also analyzed the combinations between the different indices.

**Results:**

VAI, CVAI, TyG, and LAP all have satisfactory predictive capabilities. The test capability (sensitivity and specificity) of all four indices was better or not worse than that of body mass index (BMI), homeostasis model assessment of insulin resistance (HOMA-IR) and waist circumference (WC). All four indices were effective predictors of hypogonadism at their respective cutoff values (VAI ≥ 2.284, CVAI ≥ 145.779, TyG ≥ 4.308, and LAP ≥ 59.850). Of these, LAP had the largest area under the curve (AUC, AUC = 0.852, Std. Error = 0.014, 95% CI = 0.818-0.873). However, the predictive capability of the combined indices was not significantly improved over the individual indices.

**Conclusions:**

VAI, CVAI, TyG, and LAP are sensitive indices for predicting hypogonadism in Chinese male patients with T2DM. Considering the need for concise and accurate indices in clinical practice, we suggest LAP as a commonly used index.

## 1 Introduction

Human reproductive function is strictly regulated by the metabolic state. Currently, the prevalence of obesity and diabetes is increasing worldwide (1, 2), and patients are not only at increased risk for cardiovascular events (3–5), but also for complications of the reproductive system. Studies have found that the incidence of reproductive complications in males with obesity and/or type 2 diabetes mellitus (T2DM) can range from 30% to 40%, with patients presenting with hypogonadotropic hypogonadism (6, 7). In contrast, hypogonadism is relatively rare in patients with type 1 diabetes mellitus (T1DM), suggesting that insulin resistance may play an important role (8–10). These patients present with reduced serum total testosterone (TT, recommendations from multiple societies are TT< 12 nmol/L) and clinical symptoms (10, 11), including weakness, decreased libido, erectile and ejaculatory dysfunction, etc. (12, 13). The symptoms have a negative impact on the patient’s work, life, and relationships. Meanwhile McPherson et al. found by studying male mice treated with a high-fat diet that the effects of the abnormal metabolic state on the reproductive system carried over even to their offspring raised on a normal diet (14).

There are several difficulties in the current management of male with T2DM in combination with hypogonadism. For one, the receiving physician tends to focus on renal, neurologic, and retinal complications, and rarely discusses reproductive health and refining tests for TT and other sex hormone levels (15). Secondly, in addition to TT deficiency, clinical symptoms may overlap with other complications of diabetes such as weakness, decreased libido, etc. which may be associated with poor glycemic control, vasculopathy (16), and autonomic neuropathy (17, 18). Third, the causes of hypogonadism are complex and, in clinical practice, often require the exclusion of other diseases. Obesity or diabetes results in secondary hypogonadism. It needs to be differentiated from other secondary causes (e.g., traumatic brain injury, tumors of the hypothalamus or pituitary gland, inflammatory and infectious diseases, etc.), and more importantly, primary conditions need to be excluded (e.g., congenital disorders or traumatic injuries of the testes, medications such as ketoconazole and glucocorticoid, and alcohol abuse) (19). Fourth, the appropriateness of long-term testosterone therapy for males with T2DM in the presence of hypogonadism is unclear, and the available studies have mixed results that have been summarized in review articles (20). There are also significant side effects of testosterone therapy (21, 22), which makes early detection of hypogonadism very important.

Taken together, we hope to identify clinical and or biochemical indices that can be routinely used by physicians to predict hypogonadism and to be able to recommend timely andrological evaluations. There is currently 1 trial conducted in Italy that discussed the relationship between Visceral Adiposity Index (VAI) (23), Triglyceride Glucose Index (TyG) (24), and Lipid Accumulation Product (LAP) (25), three common obesity- and lipid-related indices and TT levels in males with T2DM, and found that all three indices were negatively correlated with TT levels, and corresponding cut-off values were given (26). However, considering the significant differences in adipose tissue distribution between Caucasians and Asians (27), we therefore introduced Chinese visceral adiposity index (CVAI) (27, 28), a metric used to assess visceral adiposity dysfunction in Chinese that has been shown to be associated with obesity and multiple complications of diabetes (29–31), in the assessment of gonadal dysfunction. Similarly, we also analyzed the other three indices. To our knowledge, no studies have evaluated these four indices in Chinese males with T2DM.

To fill this gap, we clarified the strength of association of the four obesity- and lipid-related indices mentioned above in predicting hypogonadism in Chinese people, and gave their predictive cut-off values in identifying hypogonadism and analyzed the diagnostic capability of the indices when they were combined.

## 2 Materials and methods

### 2.1 Participants and methods

**Figure 1:**
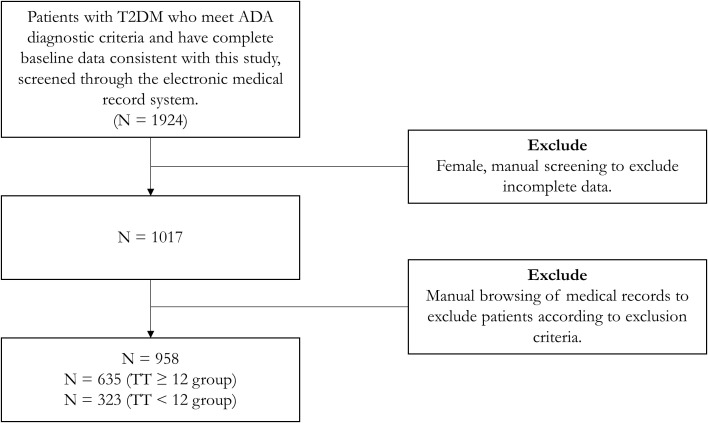
Flowchart of patient inclusion process. TT, total testosterone; T2DM, type 2 diabetes mellitus; ADA, American Diabetes Association.

We set up a database at the Affiliated Hospital of Qingdao University, from which we retrieved data on 958 male patients with T2DM who had complete sex hormone and lipid data and met the American Diabetes Association (ADA) 2023 criteria. The following were exclusion criteria:(a) Being younger than 18 or older than 70 years of age, (b) having acute complications of T2DM (e.g., diabetic ketoacidosis, hyperglycemic hyperosmolar state, etc.) or severe chronic complications of T2DM (e.g., diabetic nephropathy requiring dialysis, diabetic retinopathy with blindness or severe visual impairment, etc.), (c) malignant tumors and hematologic disorders, including after radiation or chemotherapy or surgery (d) concomitant failure of any other organ (e) infectious states or suffering from autoimmune disorders (f) hormone therapy or medications interfering with testosterone levels for any reason, and (g) any known organic hypogonadal condition (e.g., testicular inflammation, post-testicular trauma, testicular torsion, Klinefelter syndrome, Kallmann syndrome, etc.) or previous history of infertility. We demonstrated the patient inclusion process through a flowchart (**Figure 1**).

Baseline data for all subjects included age, height, weight, blood pressure, waist circumference (WC), hip circumference, duration of diabetes, as well as complication status and medication use. Body mass index (BMI) is calculated by dividing weight (kg) by the square of height (m). The circumference of the midpoint line between the lowest point of the rib cage and the upper edge of the iliac crest, measured at the end of exhalation before inhalation, defined as WC (32). And we collected blood samples from patients who had fasted for more than 8 hours to test the following items: fasting blood glucose, fasting insulin, fasting C-peptide (taking into account the use of exogenous insulin in some patients), triglycerides (TG), total cholesterol (TC), low-density lipoprotein (LDL-C), high-density lipoprotein (HDL-C), follicle-stimulating hormone (FSH), luteinizing hormone (LH), TT, estradiol (E2), creatinine, etc. We also calculated the Homeostasis Model Assessment of Insulin Resistance (HOMA-IR) to assess insulin resistance in patients, calculated as the product of fasting plasma insulin (mU/L) times fasting blood glucose (mmol/L) divided by 22.5 (33, 34). To reduce the variability of the TT assay, all tests were performed in the Central Laboratory of the Affiliated Hospital of Qingdao University (electrochemiluminescence immunoassay).

The study complied with the ethical standards of the Declaration of Helsinki (2013) and was approved by the Ethics Committee of the Affiliated Hospital of Medical College Qingdao University (QYFY WZLL 28100). All enrolled participants signed the informed consent form.

### 2.2 Indices evaluation

The four indices involved in this study can be calculated from WC (cm), TG (mmol/L), LDL-C (mmol/L), HDL-C (mmol/L), and fasting blood glucose. Below we give the formulas for VAI, CVAI, LAP and TyG (23–25, 27). Where the calculation of VAI, CVAI and LAP varies for males and females, here we give only the male (Equations 1–4).


(1) $$\begin{array}{c} VAI=\frac{\text{WC}}{39.68+1.88\times \text{BMI}}\times \frac{\text{TG}}{1.03}\times \frac{1.31}{\text{HDL}-\text{C}} \end{array}$$

(2) $$\begin{array}{c} CVAI=-267.93+0.68\times age+0.03\times BMI+4.00\times WC+22.00\times \log_{10}\text{TG}-16.32\times (\text{HDL}-\text{C}) \end{array}$$

(3) $$\begin{array}{c} LAP=\left(\text{WC}-65\right)\times TG \end{array}$$

(4) $$\begin{array}{c} TyG=\ln\frac{\text{TG }\left(\text{mg}/\text{dl}\right)\times \text{glucose}\left(\text{mg}/\text{dl}\right)}{2} \end{array}$$

### 2.3 Statistical analysis

We used SPSS software v.24.0 (SPSS IBM Corporation, Armonk, NY, USA) for statistical analysis. Quantitative variables are expressed as mean ± standard deviation or median and quartiles depending on whether they conform to a normal distribution (by the Kolmogorov-Smirnov test), and all values of qualitative variables are expressed as percentages, and comparisons between groups were made using the t-test or nonparametric Mann-Whitney U test for the quantitative variables and Fisher’s exact test or χ 2 test for the qualitative variables. Factors associated with hypogonadism were estimated using multivariate logistic regression. Considering that the indices may correlate with each other strongly, we performed calculations and presented a bivariate correlation matrix (**Supplementary Material Table 1**), and we also evaluated the covariance, giving the respective variance inflation factor (VIF) and tolerance (criteria: VIF > 10 or tolerance of approximately 0.1, **Supplementary Material Table 2**). Selected variables were not collinear. We plotted the ROC (Receiver operating characteristic) curves of the four indices to evaluate the sensitivity, specificity, and optimal cut-off values of different indices in prediction, and we also analyzed the combinations between the different indices. All statistical analyses were bilateral and P< 0.05 was considered statistically significant.

## 3 Results

### 3.1 Comparison of baseline characteristics between the two groups

**Table 1** (general characteristics of the two groups) and **Table 2** (test results and indices between the two groups) show the baseline characteristics of the participants grouped according to the TT level in a total of 958 patients, 33.72% of whom had a TT level below the hypogonadism threshold of 12 nmol/L.

**Table 1:** General characteristics of the two groups (nmol/l).

	TT ≥ 12	TT< 12	P Value
N (%)	635 (66.28%)	323 (33.72%)	
Age (year)	61.00 (54.00, 68.00)	60.00 (51.00, 69.00)	0.315
Duration of diabetes (year)	10.00 (5.00, 16.00)	10.00 (4.00, 16.00)	0.179
Height (cm)	172.00 (170.00, 176.00)	173.00 (170.00, 176.00)	0.431
Weight (kg)	75.00 (68.00, 82.00)	81.00 (73.00, 90.00)	P< 0.001
BMI (kg/m^2^)	25.10 (23.39, 27.31)	27.18 (24.93, 29.69)	P< 0.001
WC (cm)	95.00 (88.00, 101.00)	100.00 (93.00, 108.00)	P< 0.001
Hip circumference (cm)	100.00 (95.00, 104.00)	102.00 (97.00, 108.00)	P< 0.001
SBP (mmHg)	139.00 (127.00, 151.00)	140.00 (127.00, 153.00)	0.257
DBP (mmHg)	80.00 (72.00, 88.00)	80.00 (73.00, 89.00)	0.502
Tobacco intake (%)	337 (53.41)	164 (50.93)	0.469
Alcohol intake (%)	329 (52.31)	157 (48.91)	0.322
Retinopathy (%)	223 (35.12)	107 (33.13)	0.540
Nephropathy (%)	169 (26.61)	93 (28.79)	0.475
Insulin (%)	426 (67.09)	199 (61.61)	0.092
Metformin (%)	442 (69.61)	265 (82.04)	P< 0.001
α-glucosidase inhibitor (%)	428 (67.40)	220 (68.11)	0.824
DPP-4i (%)	378 (59.53)	161 (49.85)	0.004
GLP-1RA (%)	31 (4.88)	48 (14.86)	P< 0.001
Statin (%)	483 (76.06)	252 (78.02)	0.498
Fibrates (%)	15 (2.36)	22 (6.81)	0.001

TT, total testosterone; BMI, Body mass index; WC, waist circumference; SBP, systolic blood pressure; DBP, diastolic blood pressure; DPP4i, Dipeptidyl peptidase 4 inhibitors; GLP-1RA, glucagon-like peptide-1 receptor agonist.

**Table 2:** Test results and indices between the two groups (nmol/l).

	TT ≥ 12	TT< 12	P Value
Glucose (mmol/L)	6.64 (5.27, 8.51)	6.88 (5.34, 8.51)	0.826
C-peptide (ng/mL)	1.71 (1.10, 2.54)	2.29 (1.59, 3.01)	P< 0.001
Insulin (pmol/L)	6.16 (3.66, 10.36)	9.06 (5.49, 14.09)	P< 0.001
HbA1c (%)	8.05 (6.90, 9.57)	8.40 (7.20, 9.50)	0.302
HOMA-IR	0.31 (0.17, 0.56)	0.43 (0.23, 0.82)	P< 0.001
LDL-C (mmol/L)	2.55 (1.93, 3.11)	2.49 (1.87, 3.17)	0.451
HDL-C (mmol/L)	1.21 (1.06, 1.44)	1.09 (0.91, 1.27)	P< 0.001
TG (mmol/L)	1.21 (0.87, 1.86)	3.75 (1.99, 5.02)	P< 0.001
TC (mmol/L)	4.33 (3.51, 5.11)	4.20 (3.45, 5.14)	0.467
ALT (U/L)	17.00 (13.00, 25.00)	21.00 (15.00, 31.00)	P< 0.001
AST (U/L)	17.00 (14.00, 20.00)	18.00 (15.00, 24.00)	P< 0.001
mAlb (mg/L)	8.12 (4.82, 31.73)	10.90 (5.59, 34.55)	0.049
Creatinine (μmol/L)	61.00 (53.00, 71.00)	59.00 (52.00, 70.00)	0.128
eGFR (mL/min/1.73 m^2^)	115.85 (97.84, 136.69)	120.89 (99.78, 142.76)	0.110
LH (mIU/mL)	7.85 (5.90, 10.31)	7.29 (5.19, 10.35)	0.044
FSH (mIU/mL)	9.60 (6.84, 13.21)	8.92 (6.20, 14.39)	0.266
TT (nmol/l)	16.26 (14.09, 19.64)	9.25 (7.81, 10.63)	P< 0.001
E2 (pmol/L)	106.80 (83.03, 137.60)	83.29 (60.28, 109.00)	P< 0.001
LAP	35.64 (22.31, 56.99)	113.40 (73.80, 189.00)	P< 0.001
VAI	1.36 (0.87, 2.28)	4.29 (2.53, 7.09)	P< 0.001
CVAI	115.47 ± 44.37	140.29 ± 46.36	P< 0.001
TyG	4.12 (3.93, 4.34)	4.55 (4.29, 4.75)	P< 0.001

TT, total testosterone; LAP, Lipid Accumulation Product; VAI, Visceral Adiposity Index; CVAI, Chinese visceral adiposity index; TyG, Triglyceride Glucose Index; HOMA-IR, Homeostasis Model Assessment of Insulin Resistance; LDL-C, low-density lipoprotein; HDL-C, high-density lipoprotein; TG, triglycerides; TC, total cholesterol; ALT, Alanine Aminotransferase; AST, Alanine Aminotransferase; mAlb, microscale albuminuria; LH, luteinizing hormone; FSH, follicle-stimulating hormone; TT, testosterone; E2, estradiol.

Age, duration of diabetes, height, systolic blood pressure, diastolic blood pressure, smoking and alcohol consumption, comorbidities (retinopathy and nephropathy), and insulin, α-glucosidase inhibitor, and statin medications were similar between the two groups in **Table 1** (P > 0.050). However, weight, BMI, WC, and hip circumference were significantly different (P< 0.001), and the median of all four measures was greater in the TT< 12 nmol/L group than in the TT normal group.

As shown in **Table 2**, patients with TT< 12 nmol/L had similar levels of fasting glucose levels, LDL-C, TC, creatinine, and eGFR as those with TT ≥ 12 nmol/L (P > 0.050), but they had higher levels of fasting insulin, C-peptide, TG, HOMA-IR, and lower HDL-C (P< 0.001). Regarding the hypothalamic-pituitary–testicular axis, patients with TT< 12 nmol/L had lower LH but similar levels of FSH. Primarily, for the indices we want to discuss (VAI, CVAI, LAP and TyG), the levels of all four were higher in the TT< 12 nmol/L group than in the TT ≥ 12 nmol/L group (P< 0.001).

### 3.2 Logistic regression for VAI, CVAI, LAP and TyG

We performed multivariate analysis by logistic regression, after we adjusted for fasting insulin, C-peptide, TG, HDL-C, fibrates, and statins, as well as other confounders that may affect TT levels, these four indices remained predictors of a low TT (**Table 3**).

**Table 3:** Adjusted parameters of logistic regression for VAI, CVAI, LAP and TyG.

Indices	β Value	SE	Wald	P Value	OR	95% CI
BMI	0.092	0.041	4.978	0.026	1.097	1.011 - 1.189
WC	0.016	0.028	0.313	0.036	1.018	1.003 - 1.040
HOMA-IR	0.181	0.084	4.599	0.032	1.198	1.016 – 1.413
LAP	0.010	0.004	5.706	0.017	1.010	1.002 - 1.019
VAI	0.155	0.089	3.025	0.032	1.167	1.008 - 1.389
CVAI	0.007	0.005	1.413	0.035	1.009	1.002 - 1.017
TyG	0.937	0.461	4.135	0.042	2.551	1.034 - 6.292

BMI, Body mass index; WC, waist circumference; HOMA-IR, Homeostasis Model Assessment of Insulin Resistance; LAP, Lipid Accumulation Product; VAI, Visceral Adiposity Index; CVAI, Chinese visceral adiposity index; TyG, Triglyceride Glucose Index.

### 3.3 ROC curves and parameters for VAI, CVAI, LAP and TyG

**Table 4** demonstrates the parameters of the ROC curves for VAI, CVAI, LAP and TyG, all four of which were statistically significant (p<0.001), with LAP having the largest area under the curve (AUC, AUC = 0.852, Std. Error = 0.014, 95% CI = 0.818 - 0.873, and optimal cutoff value = 59.850), followed by VAI (AUC = 0.846, Std. Error = 0.014, 95% CI = 0.818 - 0.873, and optimal cutoff value = 2.284), TyG (AUC = 0.786, Std. Error = 0.016, 95% CI = 0.754 - 0.818, and optimal cutoff value = 4.308) and CVAI (AUC = 0.655, Std. Error = 0.021, 95% CI = 0.615 - 0.696, and optimal cutoff value = 145.779), all of which had test efficacies that were either better or not worse than those of WC (AUC = 0.643, Std. Error = 0.020, 95% CI = 0.604 - 0.683, and optimal cutoff value = 96.000), HOMA-IR(AUC = 0.603, Std. Error = 0.020, 95% CI =0.564 - 0.641, and optimal cutoff value = 0.370) or BMI (AUC = 0.669, Std. Error = 0.019, 95% CI =0.632 - 0.706, and optimal cutoff value = 26.704).

**Table 4:** Parameters of the ROC curves for VAI, CVAI, LAP and TyG.

Indices	AUC	SE	95% CI	P Value	Optimal cutoffs	J-Youden	Sensitivity (%)	Specificity (%)	(+) Likelihood ratio	(–) Likelihood ratio
BMI	0.669	0.019	0.632 - 0.706	< 0.001	26.704	0.278	57.59	70.24	1.93	0.60
WC	0.643	0.020	0.604 - 0.683	< 0.001	96.000	0.209	61.87	59.12	1.51	0.64
HOMA-IR	0.063	0.0198	0.564 - 0.641	< 0.001	0.370	0.194	60.13	59.28	1.48	0.67
LAP	0.852	0.014	0.825 - 0.880	< 0.001	59.850	0.619	85.24	76.66	3.65	0.19
VAI	0.846	0.014	0.818 - 0.873	< 0.001	2.284	0.558	80.74	75.05	3.24	0.26
CVAI	0.655	0.021	0.615 - 0.696	< 0.001	145.779	0.273	49.26	78.02	2.24	0.65
TyG	0.786	0.016	0.754 - 0.818	< 0.001	4.308	0.472	74.60	72.56	2.72	0.35

BMI, Body mass index; WC, waist circumference; LAP, Lipid Accumulation Product; VAI, Visceral Adiposity Index; CVAI, Chinese visceral adiposity index; TyG, Triglyceride Glucose Index.

**Figure 2:**
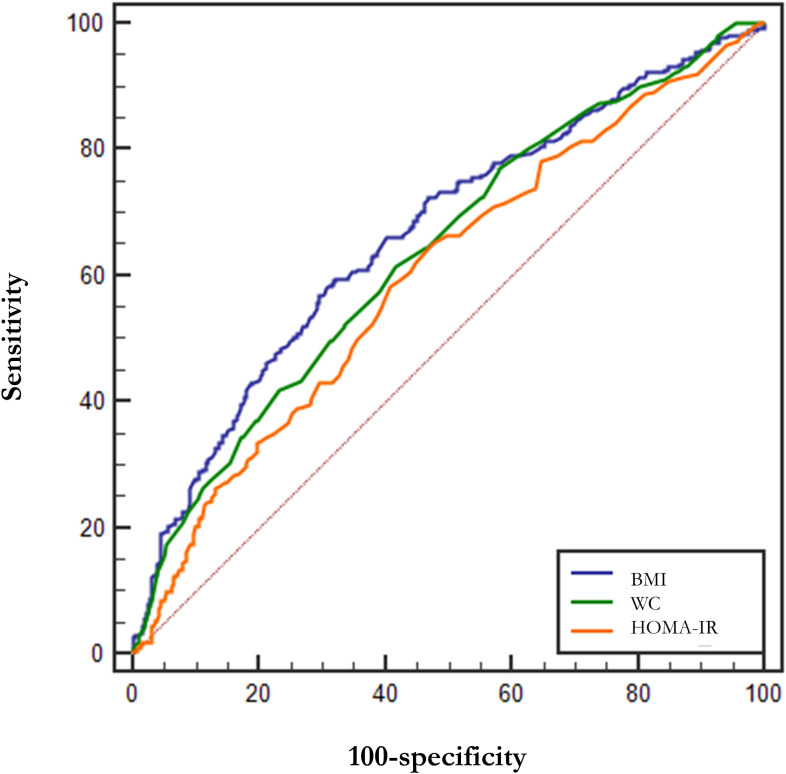
ROC curves for BMI, WC, and HOMA-IR. BMI, Body mass index; WC, waist circumference; HOMA-IR, Homeostasis Model Assessment of Insulin Resistance.

**Figure 3:**
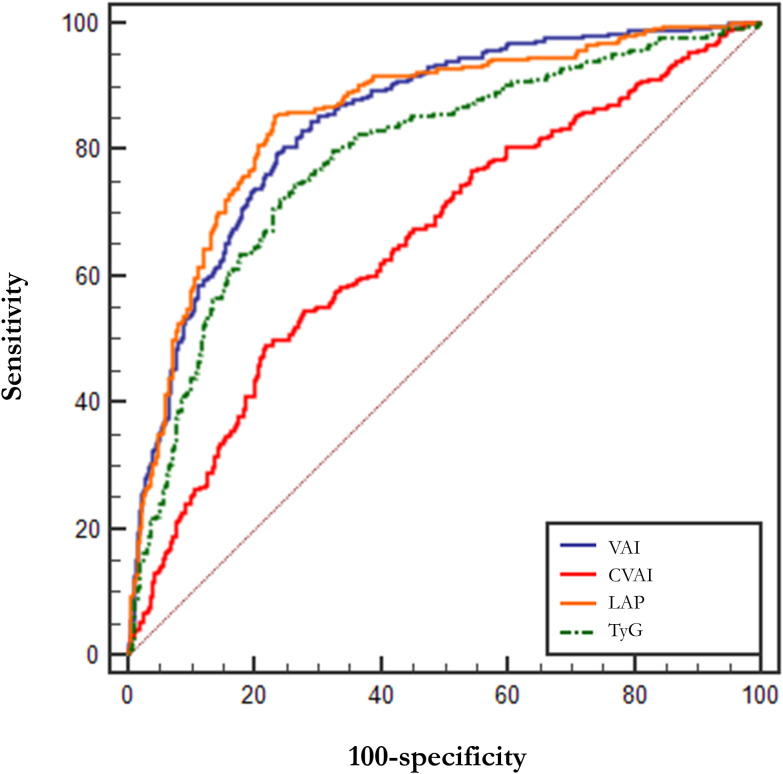
ROC curves for VAI, CVAI, LAP and TyG. LAP, Lipid Accumulation Product; VAI, Visceral Adiposity Index; CVAI, Chinese visceral adiposity index; TyG, Triglyceride Glucose Index.

**Figure 4:**
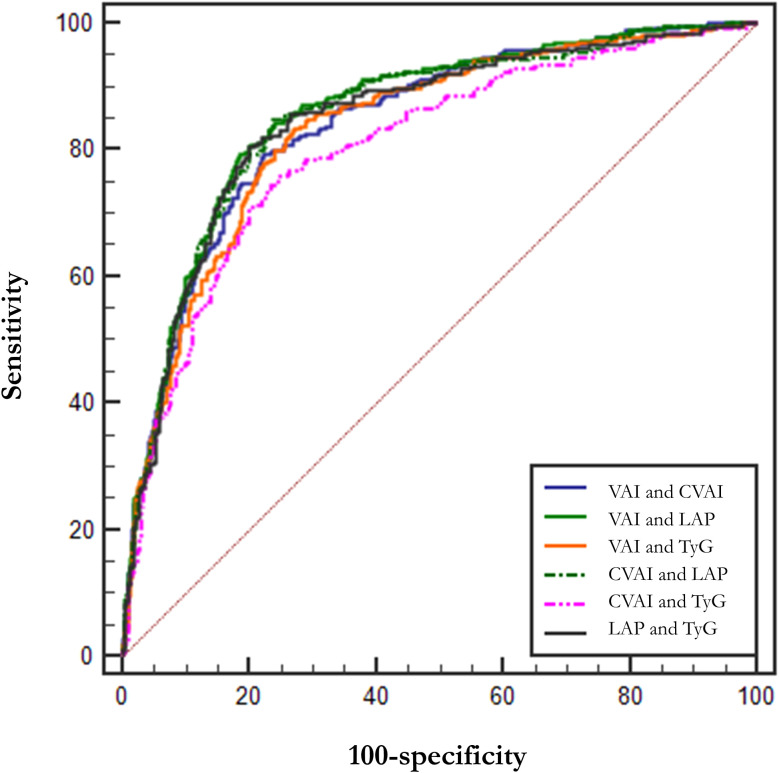
ROC curves for combined indices. LAP, Lipid Accumulation Product; VAI, Visceral Adiposity Index; CVAI, Chinese visceral adiposity index; TyG, Triglyceride Glucose Index.

In addition, we tried the combination between different indices and the results of the combinations of 2 indices are shown in **Table 5**, but the predictive capability of the combined indices was not significantly improved (no significant change in AUC or sensitivity, **Table 5**). Among them, the combination of VAI and LAP had the highest AUC (AUC = 0.857, Std. Error = 0.014, 95% CI = 0.830 - 0.884) but no increase in sensitivity. We show the results for a combination of 3 indices and a combination of 4 indices at the same time in **Supplementary Table 3**. The ROC curves for BMI, WC, and HOMA-IR are shown in **Figure 2** for reference. The ROC curves for the four individual indices are displayed in **Figure 3**, and the ROC curves for the combination of 2 indices are plotted in **Figure 4**.

**Table 5:** Parameters of the ROC curves for combinations of 2 indices.

Combination	AUC	SE	95% CI	P Value	J-Youden	Sensitivity (%)	Specificity (%)	(+) Likelihood ratio	(–) Likelihood ratio
VAI and CVAI	0.842	0.015	0.813 - 0.870	< 0.001	0.571	79.55	77.50	3.54	0.26
VAI and LAP	0.857	0.014	0.830 - 0.884	< 0.001	0.605	79.26	81.19	4.21	0.26
VAI and TyG	0.835	0.015	0.806 - 0.865	< 0.001	0.563	83.52	72.81	3.07	0.23
CVAI and LAP	0.85	0.014	0.822 - 0.878	< 0.001	0.609	84.81	76.04	3.54	0.20
CVAI and TyG	0.801	0.017	0.769 - 0.834	< 0.001	0.508	75.66	75.14	3.04	0.32
LAP and TyG	0.845	0.015	0.816 - 0.874	< 0.001	0.603	80.22	80.07	4.03	0.25

BMI, Body mass index; WC, waist circumference; LAP, Lipid Accumulation Product; VAI, Visceral Adiposity Index; CVAI, Chinese visceral adiposity index; TyG, Triglyceride Glucose Index.

## 4 Discussion

This is the first study to investigate the relationship between obesity- and lipid-related indices (VAI, CVAI, LAP and TyG) and hypogonadism in males with T2DM in Chinese population. A total of 958 male patients with T2DM were included in our study, of which 323 patients with hypogonadism, a proportion of 33.72%, was similar to the proportion in previous studies (6, 7). By analyzing this population, we found that all four indices were predictors of low TT and gave the corresponding ORs. At the same time, we made ROC curves for these four indices, confirming that they can be a more effective and reliable predictor of hypogonadism, and giving cut-off values.

Reduced TT is relatively rare in patients with type 1 diabetes, unless the patient is comorbidly obese, but are not uncommon in patients with T2DM (6, 7). A bilinear relationship between T2DM and male hypogonadism has been recognized (35), but the exact physiological mechanisms are not clear (20). It has been shown that prolonged hyperglycemia and more severe insulin resistance (both central and peripheral) affect TT synthesis and secretion (8–10), and low levels of TT are thought to exacerbate insulin resistance and even lead to diabetes (36). In our study, we found that hypogonadism was not associated with age (P = 0.315) and duration of diabetes (P = 0.179), similar to some of the previous studies (26, 37), and some of them different (38, 39). From these studies (all considering TT), age seems to be an ambiguous factor, but a cross-sectional study in Europe that included 3200 people found that TT levels appear to remain essentially stable with age until the age of 70, when elevated LH suggests functional testicular failure, while free testosterone declines progressively in the 40s and sex hormone-binding globulin (SHBG) was elevated (19, 40). Therefore, we consider that, firstly, when considering TT, age below 70 years may not be a significant influencing factor. Secondly, free testosterone, although difficult to obtain data, may be a more accurate index of gonadal function. Also to the best of our knowledge, there are no studies that discuss the relationship between age and these four indices.

In addition, we found that these patients had higher insulin and C-peptide levels, which corresponded to the more severe insulin resistance described above, as confirmed by the difference in HOMA-IR between the two groups (p< 0.001), suggesting to us that hypogonadism may be more related to diabetes control. In addition, there were differences in weight, WC, hip circumference, and BMI between the two groups (all P< 0.001), all of which have been found and repeatedly elucidated in previous studies to be associated with lower TT in males with T2DM (26, 37–39, 41), and there was a significant difference in BMI between the two groups in the present study, which fell in the obese and overweight ranges, respectively, and thus we considered BMI in the subsequent analyses.

Meanwhile, our study found a possible relationship between TT and TG and HDL-C (i.e., TT was positively correlated with HDL-C), which is consistent with many studies (26, 42, 43). This may be one of the reasons why hypogonadism is susceptible to cardiovascular disease complications (44–46), and this risk is certainly more prominent in the group of patients with T2DM. However, basic experiments yielded contradictory results, as Langer et al. found that TT upregulated the expression of scavenger receptor B1 (SR-B1) in HepG2 hepatocytes, leading to a decrease in HDL-C levels, and induced the cholesterol efflux from macrophages and retrograde transport toward the liver, and thus concluded that TT could prevent atherosclerosis (47). Taken together, the potential mechanisms underlying the association between TT and dyslipidemia in males are unclear and contradictory conclusions seem to emerge from clinical and basic studies, so further research is needed to clarify more detailed mechanisms of the relationship under the overall conditions.

VAI, CVAI, TyG, and LAP are common obesity- and lipid-related indices that reflect the distribution of fat and indirectly glyco-metabolic (48, 49). Our study also found that these indices are also sensitive predictors of hypogonadism in Chinese males with T2DM. The test capability (sensitivity and specificity) of VAI, LAP and TyG were significantly better than that of WC, HOMA-IR, and BMI, and the test capability of CVAI was not inferior to that of WC, HOMA-IR, and BMI. We also tried combinations between these four indices, but there was no significant improvement in AUC, sensitivity, and specificity. Therefore, we suggest LAP (AUC = 0.852, Std. Error = 0.014, 95% CI = 0.818 - 0.873, and optimal cutoff value = 59.850) as a commonly used index.

The relationship between low TT and obesity- and lipid-related abnormalities is currently unclear, but possible mechanisms are discussed below: First, previous studies suggested that low TT was due to increased aromatase activity in adipose tissue and increased conversion of TT to E2, which inhibits the hypothalamic-pituitary-gonadal (HPG) axis, but several more recent studies have found a positive correlation between TT and E2 (50), and our study also had similar findings (P< 0.001). And a partial explanation may be given by the study of Brüning et al. They selectively deleted insulin receptors in mice neurons, leading to hypogonadotropic hypogonadism in addition to metabolic symptoms (9). Moreover, it is known from *in vitro* experiments that insulin promotes the secretion of gonadotropin-releasing hormone (GnRH) from hypothalamic neurons, a physiological effect that is mainly mediated through phosphatidylinositol 3-kinase (PI3K) and the mitogen-activated protein kinases (MAPK) signaling pathways, two classical signaling pathways of insulin (51, 52), which activate the expression of the *c-fos* and *EGR-1* genes, and then the GnRH mRNA levels are elevated (53), and it is clear that the impedance of the signaling in the insulin-resistant state affects the expression of GnRH as well. Thus, when obesity- and lipid-related abnormalities are present, the reduction of TT and E2 suggests the possible presence of insulin resistance in neurons of the HPG axis, whereas the role of aromatase may not be significant. Second, visceral fat is also an active endocrine tissue, increased secretion of adipose-specific cytokines (e.g., leptin, IL-6, and TNF-α) in patients with obesity or abnormal adipose distribution also inhibits gonadotropin secretion, which in turn inhibits HPG axis (54, 55). This explains the lower LH in hypogonadism patients in the present study (P = 0.044) and corresponds to previous studies (56). Third, insulin resistance in abnormal fat distribution and T2DM result in a decrease in sex hormone-binding globulin (SHBG) (57), secondary to a decrease in TT.

However, our study also has limitations. First, this was a retrospective study, so we lacked the testicular examination (to assess volumes through Prader’s orchidometer), and International Index of erectile function-5 (IIEF-5), International Prostatic Symptoms Score (IPSS) and Aging Male Symptom Score (AMSS) questionnaires, and we also did not measure some important obesity-derived adipokines (e.g., Adiponectin, Leptin, Chemerin, and Nesfatin-1), which some studies suggest are associated with obesity and hypogonadism (58–60), as well as SHBG, which is used to calculate free testosterone, a more accurate reflection of actual testosterone levels. In addition, all of us recruited were hospitalized patients, so there may be some bias, such as higher age being one of them. It is also worth noting that a number of other factors can affect TT, such as statins have been found to reduce TT, particularly atorvastatin, which may be due to the fact that statins affect testicular uptake of cholesterol, the raw material for TT synthesis, and statins may lead to a reduction in SHBG (61). We also look forward to subsequent studies that incorporate relevant indices.

## 5 Conclusions

VAI, CVAI, TyG, and LAP are sensitive indicators for predicting hypogonadism in Chinese male patients with T2DM, and we give possible cutoff values, where we recommend LAP as a commonly used assessment index, and indices combinations are often unnecessary because they do not increase predictive capability. We expect to facilitate cooperation between different departments (endocrinologists and andrologists) and to contribute to the early detection and early treatment of patients with reproductive system complications.

## Data availability statement

The raw data supporting the conclusions of this article will be made available by the authors, without undue reservation.

## Ethics statement

The studies involving humans were approved by Ethics Committee of the Affiliated Hospital of Medical College Qingdao University. The studies were conducted in accordance with the local legislation and institutional requirements. The participants provided their written informed consent to participate in this study.

## Author contributions

YY: Conceptualization, Data curation, Formal analysis, Writing – original draft, Writing – review & editing. YYW: Data curation, Formal analysis, Writing – review & editing. LX: Methodology, Project administration, Writing – review & editing. WL: Data curation, Formal analysis, Writing – review & editing. YGW: Conceptualization, Supervision, Writing – review & editing.

## References

[1] Sun H Saeedi P Karuranga S Pinkepank M Ogurtsova K Duncan BB. IDF Diabetes Atlas: Global, regional and country-level diabetes prevalence estimates for 2021 and projections for 2045. Diabetes Res Clin Pract (2022) 183:109119. doi: 10.1016/j.diabres.2021.109119 34879977 PMC11057359

[2] Ng M Fleming T Robinson M Thomson B Graetz N Margono C. Global, regional, and national prevalence of overweight and obesity in children and adults during 1980-2013: a systematic analysis for the Global Burden of Disease Study 2013. Lancet (2014) 384(9945):766–81. doi: 10.1016/S0140-6736(14)60460-8 PMC4624264 24880830

[3] Sarwar N Gao P Seshasai SR Gobin R Kaptoge S Di Angelantonio E. Diabetes mellitus, fasting blood glucose concentration, and risk of vascular disease: a collaborative meta-analysis of 102 prospective studies. Lancet (2010) 375(9733):2215–22. doi: 10.1016/S0140-6736(10)60484-9 PMC2904878 20609967

[4] Kannel WB McGee DL. Diabetes and glucose tolerance as risk factors for cardiovascular disease: the Framingham study. Diabetes Care (1979) 2(2):120–6. doi: 10.2337/diacare.2.2.120 520114

[5] Cavallari I Bhatt DL Steg PG Leiter LA McGuire DK Mosenzon O. Causes and risk factors for death in diabetes: A competing-risk analysis from the SAVOR-TIMI 53 trial. J Am Coll Cardiol (2021) 77(14):1837–40. doi: 10.1016/j.jacc.2021.02.030 33832610

[6] Dandona P Dhindsa S. Update: Hypogonadotropic hypogonadism in type 2 diabetes and obesity. J Clin Endocrinol Metab (2011) 96(9):2643–51. doi: 10.1210/jc.2010-2724 PMC3167667 21896895

[7] Dhindsa S Miller MG McWhirter CL Mager DE Ghanim H Chaudhuri A. Testosterone concentrations in diabetic and nondiabetic obese men. Diabetes Care (2010) 33(6):1186–92. doi: 10.2337/dc09-1649 PMC2875421 20200299

[8] Kullmann S Heni M Hallschmid M Fritsche A Preissl H Häring HU. Brain insulin resistance at the crossroads of metabolic and cognitive disorders in humans. Physiol Rev (2016) 96(4):1169–209. doi: 10.1152/physrev.00032.2015 27489306

[9] Brüning JC Gautam D Burks DJ Gillette J Schubert M Orban PC. Role of brain insulin receptor in control of body weight and reproduction. Science (2000) 289(5487):2122–5. doi: 10.1126/science.289.5487.2122 11000114

[10] Hackett G Kirby M Rees RW Jones TH Muneer A Livingston M. The british society for sexual medicine guidelines on male adult testosterone deficiency, with statements for practice. World J Mens Health (2023) 41(3):508–37. doi: 10.5534/wjmh.221027 PMC10307648 36876744

[11] Wang C Nieschlag E Swerdloff R Behre HM Hellstrom WJ Gooren LJ. Investigation, treatment, and monitoring of late-onset hypogonadism in males: ISA, ISSAM, EAU, EAA, and ASA recommendations. J Androl (2009) 30(1):1–9. doi: 10.1530/EJE-08-0601 18772485

[12] Mostafa T Abdel-Hamid IA. Ejaculatory dysfunction in men with diabetes mellitus. World J Diabetes (2021) 12(7):954–74. doi: 10.4239/wjd.v12.i7.954 PMC8311479 34326948

[13] Zhang J Li X Cai Z Li H Yang B. Association between testosterone with type 2 diabetes in adult males, a meta-analysis and trial sequential analysis. Aging Male (2020) 23(5):607–18. doi: 10.1080/13685538.2018.1557139 30651030

[14] McPherson NO Fullston T Bakos HW Setchell BP Lane M. Obese father’s metabolic state, adiposity, and reproductive capacity indicate son’s reproductive health. Fertil Steril (2014) 101(3):865–73. doi: 10.1016/j.fertnstert.2013.12.007 24424359

[15] Foresta C Ferlin A Lenzi A Montorsi P. The great opportunity of the andrological patient: cardiovascular and metabolic risk assessment and prevention. Andrology (2017) 5(3):408–13. doi: 10.1111/andr.12342 28267892

[16] Gazzaruso C Solerte SB Pujia A Coppola A Vezzoli M Salvucci F. Erectile dysfunction as a predictor of cardiovascular events and death in diabetic patients with angiographically proven asymptomatic coronary artery disease: a potential protective role for statins and 5-phosphodiesterase inhibitors. J Am Coll Cardiol (2008) 51(21):2040–4. doi: 10.1016/j.jacc.2007.10.069 18498958

[17] Defeudis G Mazzilli R Tenuta M Rossini G Zamponi V Olana S. Erectile dysfunction and diabetes: A melting pot of circumstances and treatments. Diabetes Metab Res Rev (2022) 38(2):e3494. doi: 10.1002/dmrr.3494 34514697 PMC9286480

[18] Cellek S Cameron NE Cotter MA Muneer A. Pathophysiology of diabetic erectile dysfunction: potential contribution of vasa nervorum and advanced glycation endproducts. Int J Impot Res (2013) 25(1):1–6. doi: 10.1038/ijir.2012.30 22914567

[19] Corona G Goulis DG Huhtaniemi I Zitzmann M Toppari J Forti G. European Academy of Andrology (EAA) guidelines on investigation, treatment and monitoring of functional hypogonadism in males: Endorsing organization: European Society of Endocrinology. Andrology (2020) 8(5):970–87. doi: 10.1111/andr.12770 32026626

[20] Gianatti EJ Grossmann M. Testosterone deficiency in men with Type 2 diabetes: pathophysiology and treatment. Diabetes Med (2020) 37(2):174–86. doi: 10.1111/dme.13977 31006133

[21] Bhasin S Brito JP Cunningham GR Hayes FJ Hodis HN Matsumoto AM. Testosterone therapy in men with hypogonadism: an endocrine society clinical practice guideline. J Clin Endocrinol Metab (2018) 103(5):1715–44. doi: 10.1210/jc.2018-00229 29562364

[22] Basaria S Coviello AD Travison TG Storer TW Farwell WR Jette AM. Adverse events associated with testosterone administration. New Engl J Med (2010) 363(2):109–22. doi: 10.1056/NEJMoa1000485 PMC3440621 20592293

[23] Amato MC Giordano C Galia M Criscimanna A Vitabile S Midiri M. Visceral Adiposity Index: a reliable indicator of visceral fat function associated with cardiometabolic risk. Diabetes Care (2010) 33(4):920–2. doi: 10.2337/dc09-1825 PMC2845052 20067971

[24] Guerrero-Romero F Simental-Mendía LE González-Ortiz M Martínez-Abundis E Ramos-Zavala MG Hernández-González SO. The product of triglycerides and glucose, a simple measure of insulin sensitivity. Comparison with the euglycemic-hyperinsulinemic clamp. J Clin Endocrinol Metab (2010) 95(7):3347–51. doi: 10.1210/jc.2010-0288 20484475

[25] Kahn HS. The “lipid accumulation product” performs better than the body mass index for recognizing cardiovascular risk: a population-based comparison. BMC Cardiovasc Disord (2005) 5:26. doi: 10.1186/1471-2261-5-26 16150143 PMC1236917

[26] Caretta N Facondo P Mereu S Delbarba A Crepaldi MC Vedovato M. Cardiometabolic indices predict hypogonadism in male patients with type 2 diabetes. J Endocrinol Invest (2023) 46(3):599–608. doi: 10.1007/s40618-022-01941-0 36282472 PMC9938038

[27] Xia MF Chen Y Lin HD Ma H Li XM Aleteng Q. A indicator of visceral adipose dysfunction to evaluate metabolic health in adult Chinese. Sci Rep (2016) 6:38214. doi: 10.1038/srep38214 27905531 PMC5131270

[28] Gui J Li Y Liu H Guo LL Li J Lei Y. Obesity- and lipid-related indices as a predictor of obesity metabolic syndrome in a national cohort study. Front Public Health (2023) 11:1073824. doi: 10.3389/fpubh.2023.1073824 36875382 PMC9980350

[29] Xie X Li Q Zhang L Ren W. LIPID ACCUMULATION PRODUCT, VISCERAL ADIPOSITY INDEX, AND CHINESE VISCERAL ADIPOSITY INDEX AS MARKERS OF CARDIOMETABOLIC RISK IN ADULT GROWTH HORMONE DEFICIENCY PATIENTS: A CROSS-SECTIONAL STUDY. Endocr Pract (2018) 24(1):33–9. doi: 10.4158/EP-2017-0007 29144802

[30] Qiao T Luo T Pei H Yimingniyazi B Aili D Aimudula A. Association between abdominal obesity indices and risk of cardiovascular events in Chinese populations with type 2 diabetes: a prospective cohort study. Cardiovasc Diabetol (2022) 21(1):225. doi: 10.1186/s12933-022-01670-x 36320060 PMC9628026

[31] Li X Li HY Yu ZW Zhang YT Tong XW Gao XY. Association among lipid accumulation product, chinese visceral obesity index and diabetic retinopathy in patients with type 2 diabetes: A cross-sectional study. Diabetes Metab Syndr Obes (2021) 14:4971–9. doi: 10.2147/DMSO.S348195 PMC8721021 35002269

[32] Zhang L Yang L Wang C Yuan T Zhang D Wei H. Mediator or moderator? The role of obesity in the association between age at menarche and blood pressure in middle-aged and elderly Chinese: a population-based cross-sectional study. BMJ Open (2022) 12(5):e051486. doi: 10.1136/bmjopen-2021-051486 PMC9137347 35618334

[33] Matthews DR Hosker JP Rudenski AS Naylor BA Treacher DF Turner RC. Homeostasis model assessment: insulin resistance and beta-cell function from fasting plasma glucose and insulin concentrations in man. Diabetologia (1985) 28(7):412–9. doi: 10.1007/BF00280883 3899825

[34] Ranjan A Choubey M Yada T Krishna A. Nesfatin-1 ameliorates type-2 diabetes-associated reproductive dysfunction in male mice. J Endocrinol Invest (2020) 43(4):515–28. doi: 10.1007/s40618-019-01136-0 31691259

[35] Rao PM Kelly DM Jones TH. Testosterone and insulin resistance in the metabolic syndrome and T2DM in men. Nat Rev Endocrinol (2013) 9(8):479–93. doi: 10.1038/nrendo.2013.122 23797822

[36] Tsai EC Matsumoto AM Fujimoto WY Boyko EJ. Association of bioavailable, free, and total testosterone with insulin resistance: influence of sex hormone-binding globulin and body fat. Diabetes Care (2004) 27(4):861–8. doi: 10.2337/diacare.27.4.861 15047639

[37] Zheng R Cao L Cao W Chu X Hu Y Zhang H. Risk factors for hypogonadism in male patients with type 2 diabetes. J Diabetes Res (2016) 2016:5162167. doi: 10.1155/2016/5162167 27006953 PMC4781970

[38] Al Hayek AA Khader YS Jafal S Khawaja N Robert AA Ajlouni K. Prevalence of low testosterone levels in men with type 2 diabetes mellitus: a cross-sectional study. J Family Community Med (2013) 20(3):179–86. doi: 10.4103/2230-8229.122006 PMC3957172 24672276

[39] Kapoor D Aldred H Clark S Channer KS Jones TH. Clinical and biochemical assessment of hypogonadism in men with type 2 diabetes: correlations with bioavailable testosterone and visceral adiposity. Diabetes Care (2007) 30(4):911–7. doi: 10.2337/dc06-1426 17392552

[40] Wu FC Tajar A Pye SR Silman AJ Finn JD O’Neill TW. Hypothalamic-pituitary-testicular axis disruptions in older men are differentially linked to age and modifiable risk factors: the European Male Aging Study. J Clin Endocrinol Metab (2008) 93(7):2737–45. doi: 10.1210/jc.2007-1972 18270261

[41] Su M Wei H Chen L Guan Y Dong W Zhao M. The impact of visceral adiposity on testosterone levels in american adult men: A cross-sectional analysis. Med Sci Monit (2023) 29:e941394. doi: 10.12659/MSM.941394 37634076 PMC10469406

[42] Zhang N Zhang H Zhang X Zhang B Wang F Wang C. The relationship between endogenous testosterone and lipid profile in middle-aged and elderly Chinese men. Eur J Endocrinol (2014) 170(4):487–94. doi: 10.1530/EJE-13-0802 24394726

[43] Akishita M Fukai S Hashimoto M Kameyama Y Nomura K Nakamura T. Association of low testosterone with metabolic syndrome and its components in middle-aged Japanese men. Hypertens Res (2010) 33(6):587–91. doi: 10.1038/hr.2010.43 20339372

[44] Gandaglia G Briganti A Jackson G Kloner RA Montorsi F Montorsi P. A systematic review of the association between erectile dysfunction and cardiovascular disease. Eur Urol (2014) 65(5):968–78. doi: 10.1016/j.eururo.2013.08.023 24011423

[45] Montorsi P Ravagnani PM Galli S Rotatori F Veglia F Briganti A. Association between erectile dysfunction and coronary artery disease. Role of coronary clinical presentation and extent of coronary vessels involvement: the COBRA trial. Eur Heart J (2006) 27(22):2632–9. doi: 10.1093/eurheartj/ehl142 16854949

[46] Vlachopoulos C Jackson G Stefanadis C Montorsi P. Erectile dysfunction in the cardiovascular patient. Eur Heart J (2013) 34(27):2034–46. doi: 10.1093/eurheartj/eht112 23616415

[47] Langer C Gansz B Goepfert C Engel T Uehara Y von Dehn G. Testosterone up-regulates scavenger receptor BI and stimulates cholesterol efflux from macrophages. Biochem Biophys Res Commun (2002) 296(5):1051–7. doi: 10.1016/S0006-291X(02)02038-7 12207878

[48] Ahn N Baumeister SE Amann U Rathmann W Peters A Huth C. Visceral adiposity index (VAI), lipid accumulation product (LAP), and product of triglycerides and glucose (TyG) to discriminate prediabetes and diabetes. Sci Rep (2019) 9(1):9693. doi: 10.1038/s41598-019-46187-8 31273286 PMC6609728

[49] Golabi S Ajloo S Maghsoudi F Adelipour M Naghashpour M. Associations between traditional and non-traditional anthropometric indices and cardiometabolic risk factors among inpatients with type 2 diabetes mellitus: a cross-sectional study. J Int Med Res (2021) 49(10):3000605211049960. doi: 10.1177/03000605211049960 34657502 PMC8524710

[50] Dhindsa S Furlanetto R Vora M Ghanim H Chaudhuri A Dandona P. Low estradiol concentrations in men with subnormal testosterone concentrations and type 2 diabetes. Diabetes Care (2011) 34(8):1854–9. doi: 10.2337/dc11-0208 PMC3142021 21715518

[51] Harada S Smith RM Smith JA White MF Jarett L. Insulin-induced egr-1 and c-fos expression in 32D cells requires insulin receptor, Shc, and mitogen-activated protein kinase, but not insulin receptor substrate-1 and phosphatidylinositol 3-kinase activation. J Biol Chem (1996) 271(47):30222–6. doi: 10.1074/jbc.271.47.30222 8939974

[52] Keeton AB Bortoff KD Bennett WL Franklin JL Venable DY Messina JL. Insulin-regulated expression of Egr-1 and Krox20: dependence on ERK1/2 and interaction with p38 and PI3-kinase pathways. Endocrinology (2003) 144(12):5402–10. doi: 10.1210/en.2003-0592 12970165

[53] Salvi R Castillo E Voirol MJ Glauser M Rey JP Gaillard RC. Gonadotropin-releasing hormone-expressing neurons immortalized conditionally are activated by insulin: implication of the mitogen-activated protein kinase pathway. Endocrinology (2006) 147(2):816–26. doi: 10.1210/en.2005-0728 16293665

[54] Grossmann M. Hypogonadism and male obesity: Focus on unresolved questions. Clin Endocrinol (Oxf) (2018) 89(1):11–21. doi: 10.1111/cen.13723 29683196

[55] Makki K Froguel P Wolowczuk I. Adipose tissue in obesity-related inflammation and insulin resistance: cells, cytokines, and chemokines. ISRN Inflamm (2013) 2013:139239. doi: 10.1155/2013/139239 24455420 PMC3881510

[56] Vermeulen A Kaufman JM Deslypere JP Thomas G. Attenuated luteinizing hormone (LH) pulse amplitude but normal LH pulse frequency, and its relation to plasma androgens in hypogonadism of obese men. J Clin Endocrinol Metab (1993) 76(5):1140–6. doi: 10.1210/jcem.76.5.8496304 8496304

[57] Rajala UM Keinänen-Kiukaanniemi SM Hirsso PK Jokelainen JJ Laakso MA Hiltunen LA. Associations of total testosterone and sex hormone-binding globulin levels with insulin sensitivity in middle-aged Finnish men. Diabetes Care (2007) 30(4):e13. doi: 10.2337/dc06-1979 17392528

[58] Choubey M Ranjan A Bora PS Krishna A. Protective role of adiponectin against testicular impairment in high-fat diet/streptozotocin-induced type 2 diabetic mice. Biochimie (2020) 168:41–52. doi: 10.1016/j.biochi.2019.10.014 31676315

[59] Estienne A Bongrani A Reverchon M Ramé C Ducluzeau PH Froment P. Involvement of novel adipokines, chemerin, visfatin, resistin and apelin in reproductive functions in normal and pathological conditions in humans and animal models. Int J Mol Sci (2019) 20(18). doi: 10.3390/ijms20184431 PMC6769682 31505789

[60] Singh A Choubey M Bora P Krishna A. Adiponectin and chemerin: contrary adipokines in regulating reproduction and metabolic disorders. Reprod Sci (2018) 25(10):1462–73. doi: 10.1177/1933719118770547 29669464

[61] Stanworth RD Kapoor D Channer KS Jones TH. Statin therapy is associated with lower total but not bioavailable or free testosterone in men with type 2 diabetes. Diabetes Care (2009) 32(4):541–6. doi: 10.2337/dc08-1183 PMC2660443 19114614

