# Supplementary material for: Combined obesity- and lipid-related indices are associated with hypogonadism in Chinese male patients with type 2 diabetes: a cross-sectional study
Source: Front Endocrinol (Lausanne). 2024 Jan 8;14:1319582. doi: 10.3389/fendo.2023.1319582 (PMC10801025; doi:10.3389/fendo.2023.1319582)
Supplement: Supplementary file 1 [file 1319582_Table_1.docx]

Supplementary Material

# Supplementary Tables

## Supplementary Table 1

**Supplementary Table 1 Spearman binary correlation matrix between four combined obesity- and lipid-related indices**

|  | LAP | VAI | CVAI | TyG |
| --- | --- | --- | --- | --- |
| LAP | 1 |  |  |  |
| VAI | 0.926** | 1 |  |  |
| CVAI | 0.548** | 0.374** | 1 |  |
| TyG | 0.839** | 0.854** | 0.243** | 1 |

**P < 0.01. LAP, Lipid Accumulation Product; VAI, Visceral Adiposity Index; CVAI, Chinese visceral adiposity index; TyG, Triglyceride Glucose Index.

## Supplementary Table 2

**Supplementary Table 2 Variance inflation factor and tolerance four combined obesity- and lipid-related indices**

| **Indices** | **Tolerances** | **VIF** |
| --- | --- | --- |
| LAP | 0.301 | 3.323 |
| VAI | 0.533 | 1.877 |
| CVAI | 0.820 | 1.219 |
| TyG | 0.516 | 1.937 |

LAP, Lipid Accumulation Product; VAI, Visceral Adiposity Index; CVAI, Chinese visceral adiposity index; TyG, Triglyceride Glucose Index. VIF, Variance Inflation Factor.

## Supplementary Table 3

**Supplementary Table 3 Parameters of the ROC curves for combinations of 3 and 4 indices.**

| **Combination** | **AUC** | **SE** | **95% CI** | **P Value** | **J-Youden** | **Sensitivity (%)** | **Specificity (%)** | **(+) Likelihood ratio** | **(-) Likelihood ratio** |
| --- | --- | --- | --- | --- | --- | --- | --- | --- | --- |
| VAI, CVAI and LAP | 0.852 | 0.014 | 0.825 - 0.880 | ＜ 0.001 | 0.602 | 81.04 | 79.13 | 3.88 | 0.24 |
| VAI, CVAI and TyG | 0.838 | 0.015 | 0.809 - 0.868 | ＜ 0.001 | 0.566 | 77.07 | 79.52 | 3.76 | 0.29 |
| VAI, LAP and TyG | 0.849 | 0.015 | 0.821 - 0.878 | ＜ 0.001 | 0.603 | 80.52 | 79.74 | 3.98 | 0.24 |
| CVAI, LAP and TyG | 0.841 | 0.015 | 0.812 - 0.871 | ＜ 0.001 | 0.595 | 80.15 | 79.31 | 3.87 | 0.25 |
| All 4 indices | 0.845 | 0.015 | 0.816 - 0.874 | ＜ 0.001 | 0.586 | 81.95 | 76.60 | 3.50 | 0.24 |

LAP, Lipid Accumulation Product; VAI, Visceral Adiposity Index; CVAI, Chinese visceral adiposity index; TyG, Triglyceride Glucose Index. VIF, Variance Inflation Factor.
